# Monitoring human cytomegalovirus infection with nested PCR: comparison of positive rates in plasma and leukocytes and with quantitative PCR

**DOI:** 10.1186/1743-422X-7-73

**Published:** 2010-04-15

**Authors:** Shu Zhang, Yi-Hua Zhou, Lei Li, Yali Hu

**Affiliations:** 1 Department of Obstetrics and Gynecology, Nanjing Drum Tower Hospital, Nanjing University Medical School, Nanjing, China; 2 Departments of Infectious Diseases and Laboratory Medicine, Nanjing Drum Tower Hospital, Nanjing University Medical School, Nanjing, China; 3 Jiangsu Key Laboratory for Molecular Medicine, Nanjing University Medical School, Nanjing, China

## Abstract

**Background:**

Human cytomegalovirus (HCMV) infection poses a significant health threat to immunocompromised individuals. Here we performed this study to set up a highly sensitive nested PCR method applicable for detecting HCMV infection in high-risk individuals. In this work, 106 blood specimens from 66 patients with potential HCMV infection were obtained. Total DNA was extracted separately from plasma and peripheral blood leukocytes (PBL) of each sample. HCMV DNA was detected in parallel by nested PCR and quantitative real-time PCR (qRT-PCR), and the results were compared.

**Results:**

Serial dilution test revealed that the detection limit of nested PCR was 180 copies/ml. The nested PCR showed a higher positive rate than qRT-PCR (34.9% vs. 12.3%, p < 0.001). The positive rate of nested PCR based on PBL DNA was significantly higher than that based on plasma DNA (34.9% vs. 18.9%, p = 0.002). Of the 14 patients with serial samples, 11 were positive for HCMV DNA in PBL while only 7 were positive in plasma. Moreover, for each patient, nested PCR using PBL DNA also detected more positive samples than that using plasma DNA.

**Conclusion:**

Combined use of nested PCR with PBL DNA is highly sensitive in defining HCMV infection. This assay is particularly useful in the case of quantification not essential.

## Background

Human cytomegalovirus (HCMV), an opportunistic pathogen, is ubiquitously distributed in human population. The seroprevalence of HCMV in adults ranges from 55% [1] in developed countries to as high as over 90% [2,3] in developing countries like China. In immunocompetent persons, HCMV usually just causes a latent infection with few obvious lesions, but it poses a significant health threat to immunocompromised individuals, such as allograft organ transplant recipients, AIDS patients and chemotherapy recipients. Additionally, the viral intrauterine transmission to fetus can lead to stillbirth, abortion, and mental retardation. Thus the availability of a highly sensitive test capable of detecting HCMV infection for these populations is of great significance.

Although the detection of pp65 antigen on peripheral blood leukocytes provides early diagnosis, its sensitivity, 16-68% as reported [4,5], is not so satisfied. Polymerase chain reaction (PCR) has been proved to be a sensitive and effective technique in defining HCMV infection. Quantitative real-time PCR (qRT-PCR) is widely applied for its preponderance in quantifying the viral load and reducing the probability of contamination by reacting in a closed system. Nested PCR uses two amplification rounds with the two pairs of "external" and "internal" primers, hence the sensitivity and specificity are improved significantly. However, it is still a subject being debated to determine which of the two is more sensitive and suitable for monitoring HCMV infection, since the results of many relevant reports are contradictory [6-10].

In addition to detection assay, it has been documented that different blood components also affect the positive rate of HCMV infection [11,12]. Which type of blood fractions, peripheral blood leukocytes (PBL) or plasma, is optimal? There has been no consensus yet. Some reports [12-14] suggest that leukocyte-based tests are superior for detecting HCMV DNA. On the other hand, several studies show that plasma/serum positivity is more correlated with active infection and the processing of plasma is easier [15,16].

The aim of our study was to set up a highly sensitive assay applicable to detecting HCMV infection in high-risk patients. In the present study, two PCR techniques, qRT-PCR and nested PCR, and different materials, plasma and PBL, were compared in detecting HCMV DNA. The serological assays including IgM, IgG, and IgG avidity index (AI) tests were also performed to verify the results of PCR, since Lutz et al [17,18] have proposed that a low IgG AI is still meaningful in identifying primary infection even for immunosuppressed patients.

## Results

### The specificity and sensitivity of nested PCR

To determine the specificity and sensitivity of nested PCR, we detected HCMV DNA in one viral culture supernatant, 4 plasma specimens from the confirmed patients, and 4 plasmas from healthy adults. As expected, HCMV DNA was positive in the culture supernatant and in the 4 patients' plasma, while none of the 4 healthy persons' specimen presented a specific 293-bp fragment (data not shown), suggesting the reliable specificity of this nested PCR. One positive plasma sample, quantified by qRT-PCR, underwent 2-fold serial dilution and amplification by nested PCR. The HCMV DNA was still detected by nested PCR even though the viral load was as low as 180 copies/ml, indicating that nested PCR is more sensitive than qRT-PCR, in which the detection limit was 500 copies/ml according to the manufacture's instructions.

### The positive rate of HCMV DNA in plasma and PBL detected by qRT-PCR and nested PCR

To evaluate the detection efficacy of different PCR techniques, we simultaneously performed nested PCR and qRT-PCR for the 106 specimens from 66 patients. To verify the contribution of different materials to the positive rate, we also detected the viral DNA in both the plasma and PBL of each specimen. As shown in Table 1, all the 13 positive samples by qRT-PCR were also positive by nested PCR both with plasma and PBL. With nested PCR, 18 of 20 positive samples by plasma DNA were also positive by leukocyte DNA; moreover, 19 negative samples by plasma DNA were positive by leukocyte DNA. Together, the sensitivity of nested PCR was higher than that of qRT-PCR (34.9% vs. 12.3%, p < 0.001), and PBL was superior to plasma with higher sensitivity (34.9% vs. 18.9%, p = 0.002).

**Table 1:** Positive rate of HCMV DNA in plasma and PBL by qRT-PCR and nested PCR

Technique with material No. of positive cases (%) (N = 106)		qRT-PCR with PBL 13 (12.3%)		Nested PCR with plasma 20 (18.9%^a^)	
			
		Positive	Negative	Positive	Negative
Nested PCR with PBL 37 (34.9%^b, c^)	Positive	13	24	18	19
	Negative	0	99	2	67

^a^Compared to the positive rate by qRT-PCR with PBL, p = 0.016.

^b^Compared to the positive rate by qRT-PCR with PBL, p < 0.001.

^c^Compared to the positive rate by nested PCR with plasma, p = 0.002.

Agreement in the three processes (qRT-PCR with PBL, nested PCR with plasma, and nested PCR with PBL) was observed in 80 (75.5%) samples, including 13 positive samples and 67 negative samples. When nested PCR with PBL was considered as the reference technique, the sensitivity, specificity, positive predictive value, and negative predictive value of nested PCR with plasma and qRT-PCR with PBL, and their respective concordance to nested PCR with PBL were shown in Table 2. The three techniques had similar specificity and positive predictive value, yet nested PCR with PBL exhibited the highest sensitivity and negative predictive value.

**Table 2:** Sensitivity, specificity and predictive values of nested PCR with plasma and qRT-PCR with PBL

Process	Sensitivity (%)	Specificity (%)	Positive predictive value (%)	Negative predictive value (%)	Concordance to nested PCR with PBL (%)
qRT-PCR with PBL	35.1	100	100	74.2	77.4
Nested PCR with plasma	48.6	97.1	90.0	77.9	80.2
Nested PCR with PBL^a^	100	100	100	100	--

^a^Nested PCR with PBL was considered as the reference technique in detecting HCMV DNA.

Of the 14 cases with serial samples, 7 patients each presented at least one positive specimen by nested PCR with plasma. However, when we chose PBL as the detection material, additional 4 patients were found to be positive (Table 3). Moreover, for each individual, nested PCR with PBL detected more positive samples than that using plasma DNA. These results suggested that PBL was the preferred material in improving the sensitivity.

**Table 3:** Positive frequency of serial samples by qRT-PCR with PBL, nested PCR with PBL, and nested PCR with plasma

	Positive/Total		
	
	qRT-PCR with PBL	Nested PCR with PBL	Nested PCR with plasma
A (bone marrow transplant recipient)	3/8	5/8	3/8
B (acute myeloblastic leukemia)	0/6	3/6	0/6
C (chronic myeloblastic leukemia)	0/3	2/3	0/3
D (chronic myeloblastic leukemia)	2/8	2/8	2/8
E (systemic lupus erythematosus)	1/3	2/3	1/3
F (chickenpox)	3/3	3/3	3/3
G (acute non-lymphocytic leukemia)	0/6	2/6	0/6
H (interstitial pneumonia)	1/2	2/2	1/2
I (acute non-lymphocytic leukemia)	0/4	0/4	0/4
J (fever of undetermined origin)	0/3	0/3	0/3
K (fever of undetermined origin)	1/2	2/2	1/2
L (chronic renal failure)	1/2	2/2	2/2
M (acute myelomonocytic leukemia)	0/2	1/2	0/2
N (acute non-lymphocytic leukemia)	0/2	0/2	0/2

### The consistency of nested PCR and serological assays

Among the 66 patients with single samples, 5 had no sufficient plasma for further IgG, IgM and IgG AI detection. Thus 61 patients underwent the serological tests; all were positive for HCMV IgG. For patients with serial samples, the criterion for primary infection was: (1) all the consecutive specimens had an AI value < 30%, or (2) the AI of the initial specimen was < 30%, then it increased to > 30% in subsequent samples.

According to the results of nested PCR with either plasma or PBL, the 61 subjects were divided into positive group (24 patients) and negative group (37 patients). Considering the serological evidence for recent infection in the nested PCR positive group, 5 patients presented positive IgM only, 6 had low AI only, and 5 were positive for IgM and had low AI. On the other hand, in the nested PCR negative group, 2 were solely IgM positive, 2 had low AI, and only 1 showed for both (Table 4). The results of serological assays and nested PCR were consistent in 78.7% patients, including 16 concordant positive patients and 32 concordant negative patients. Compared with those negative in nested PCR, the nested PCR positive group had significant higher probability (66.7% vs. 13.5%, p < 0.01) to be IgM positive and/or to have low AI.

**Table 4:** Number of patients with IgM+/low AI in nested PCR (+) and nested PCR (--) groups

Group (N = 61)	IgM+ only	AI < 30% only	IgM+ and AI < 30%	Total
Nested PCR + (24)	5 (20.8%)	6 (25%)	5 (20.8%)	16 (66.7%^a^)
Nested PCR -- (37)	2 (5.4%)	2 (5.4%)	1 (2.7%)	5 (13.5%)

^a ^Compared to the nested PCR (--) group, p < 0.01.

## Discussion

In the present study, we found that nested PCR detected more samples positive for HCMV DNA than qRT-PCR, and nested PCR using PBL DNA had the highest positive rate. Therefore, nested PCR based on PBL DNA is the most sensitive method for detecting HCMV DNA.

False positive caused by cross contamination is a main problem of nested PCR. To avoid such cross contamination, we strictly adopted precautionary measures [19], such as processing meticulously, introducing negative controls, extracting and amplifying the DNA in different rooms, and changing gloves frequently. Additionally, the positives of nested PCR were confirmed to some extent by the serological assays (Table 4). Furthermore, we performed another nested PCR using a different set of primers and obtained the identical results (data not shown). Therefore, the high sensitivity of this nested PCR in detecting HCMV DNA observed in the study is reliable.

Our findings are in agreement with the previous observation that nested PCR is more sensitive than qRT-PCR [6,7]. By contrast, several studies [8,9] reported the opposite results. Additionally, the similar sensitivity or discordant results between qRT-PCR and nested PCR have also been documented by other investigators [10,20,21]. One reason for the situation of "different study, different result" may be different patient groups investigated by different studies. Mhiri et al [22] have suggested that the performance of diagnostic tests may be affected by transplant patients' different immunological responses to different transplant (renal, bone marrow, etc.). However, in the present study, we evaluated all the patients as a whole instead of dividing them into subgroups according to their primary diseases, because we aimed to define a routine test competent enough for most, if not all, patients. Actually, it is impracticable to change the detection for every specific disease during one laboratory's daily work. In addition to the targeted population, other factors such as the PCR primers [23,24] and the DNA extraction technique [25] may also influence the amplification efficacy. As Bastien et al [26] conclude that the PCR is not an isolated technique; instead, it involves a range of techniques, which might affect the outcome depending on a variety of factors. For this study, we believe that more reaction cycles in nested PCR, almost twice of that in qRT-PCR, is the main explanation for the higher sensitivity.

The detection capacity of the qRT-PCR in this investigation appears to be inferior to that of some recorded qRT-PCR [27,28]. However, a recent multi-center research [29] has indicated that various HCMV DNA quantitative assays, including both commercial reagents and laboratory-developed assays, are generally not sensitive in detecting viral loads lower than 1000 copies/ml, irrespective of their reported detection limits. Another limitation of this study is that the sample size was relatively small; nevertheless, this study contained prospective serial samples in which HCMV DNA was detected more frequently by nested PCR than by qRT-PCR (Table 3).

Although qRT-PCR has advantages in quantification, automation, and time savings [30,31], nested PCR is still clinically significant in some settings. Nested PCR is suitable for small number of samples, especially for situations in which quantification is not essential. For examples, detection of HCMV DNA in fetuses or newborns is sufficiently to diagnose an ongoing active infection. Additionally, nested PCR does offer benefits for developing countries because of its low cost.

With appropriate materials adopted in nested PCR, our results demonstrated that positive rate of HCMV DNA in PBL was much higher than that in plasma (34.9% vs. 18.9%, p = 0.002, Table 1), which is in accordance with the reported data [13,14,32], although similar sensitivities of plasma and PBL were referred in Ye et al's [33] and Banan et al's reports [34]. Two reasons may contribute to the higher sensitivity of using PBL DNA: (1) the concentration of DNA extracted from PBL should be much higher than that from 200 μl plasma, and (2) with the biological characteristics of latent infection, HCMV may be harbored in PBL [35]; when the virus in PBL replicates at a low level, very a few viruses are released into plasma, causing the lower sensitivity of using plasma DNA.

It is considered that positive DNA in serum/plasma is connected more closely with symptomatic infection [15,16], because serum/plasma DNA reflects disruption of host cells caused by active viral replication. In other words, it seems that PCR using PBL DNA is not suitable for diagnosing active CMV infection, because healthy adults with the latent infection may harbor CMV in their leukocytes. Nevertheless, for immunosuppression patients, the risk of the so-called "latency" to be activated is highly possible. Therefore the early detection of HCMV DNA is especially important for reminding clinicians to be aware of the possible reactivation and to carefully follow-up viral load kinetics. As indicated by relevant studies [31,32,36], monitoring HCMV DNA in PBL is helpful in anticipating viral replication. In our study, four patients were negative for HCMV DNA in plasma but positive for DNA in PBL (Table 3), indicating the high sensitivity by nested PCR with PBL. On the other hand, the negative result detected by nested PCR with PBL may be helpful in ruling out HCMV infection, since its negative predictive value is very high (Table 2).

## Conclusion

In summary, we confirmed that nested PCR based on PBL-derived DNA has the highest sensitivity in defining HCMV infection. This technique may be particularly suitable for small size of samples demanding highly sensitive detection, such as: (1) monitoring HCMV infection in immunocompromised patients; (2) diagnosing congenital or prenatal infection of HCMV, because the presence of HCMV DNA in fetal leucocytes is undoubtedly due to congenital infection; and (3) screening blood donors for the HCMV-infected PBL to prevent transfusion-transmitted CMV infection.

## Materials and methods

### Patients and samples

One hundred and six peripheral blood samples were obtained from 66 patients with suspected HCMV infection, 14 of who had serial specimens. Their diagnoses included leukemia, bone marrow transplant recipients, interstitial pneumonia, fever of undetermined origin, and others. Approximately 1.0 ml of EDTA-anticoagulated whole blood from each patient was collected and centrifuged at 2500 rpm for 5 min and the plasma was isolated. The remaining blood cells were treated with 5 volumes of red blood cell lysis buffer (NH_4_Cl 139.6 mM, Tris 16.96 mM, pH 7.2) for 10 min, and centrifuged. After an additional lysis, the PBL was resuspended in 200 μl normal saline. The plasma and PBL were stored at -20°C.

### Nested PCR

Total DNA was extracted as described previously [37]. Briefly, 200 μl plasma or 200 μl PBL suspension was mixed with 300 μl proteinase K (0.2 mg/ml) in a buffer containing 100 mM NaCl, 10 mM Tris-Cl, 25 mM EDTA, and 0.5% SDS (pH 8.0). After incubation at 55°C for 3 hours, DNA was extracted twice by equal volume of phenol/chloroform and subsequently precipitated by ethanol. The DNA was dissolved in 20 μl TE buffer (10 mM Tris, 1 mM EDTA).

The first round of PCR was carried out in a 50 μl-volume containing 1.5 μl of each external upstream and downstream primers (10 mM), 2 U of Taq DNA polymerase, and 4 μl of DNA template (equivalent to DNA extracted from 40 μl plasma or from 4-10 × 10^5 ^PBL). The primers, termed the IE primers, were specific for the fourth exon of the immediate early (IE) gene of HCMV [38]. The sequences were as follows: the external primers 1a 5'-GGTCACTAGTGACGCTTGTATGATGA-3', 1b 5'-GATAGTCGCGGGTACAGGGGACTCT-3'; the internal primers 2a 5'-AAGTGAGTTCTGTCGGGTGCT-3', 2b 5'-GTGACACCAGAGAATCAGA GGA-3'. After initial denature at 94°C for 5 min, 35 cycles of DNA amplification were performed (94°C for 30 sec, 58°C for 30 sec, 72°C for 60 sec), followed by terminal extension at 72°C for 5 min. Subsequently, 2 μl of first round products was subjected to second round PCR for 30 cycles in 25 μl mixture, containing 1 μl (10 mM) of each internal HCMV-specific primers and 1 U of Taq polymerase. During the second round, the extension time was 50 sec.

After amplification, 5 μl PCR products were electrophoresed on 1.5% agarose gel, stained with ethidium bromide, and photographed on an ultraviolet light transilluminator. A reliable positive result was documented when both the sample and positive control presented a band corresponding to a 293-bp DNA fragment while the negative control did not. During the PCR process, precautions [19] were strictly carried out to avoid the cross contamination.

### qRT-PCR

The qRT-PCR was carried out using a commercially available HCMV fluorescence quantitative PCR diagnostic kit (DaAn Gene Co., Ltd., Zhongshan University, China), as described by Sun Z et al [39]. The kit contains reagents and enzymes for the specific amplification of a 86-bp region of the IE1 gene of HCMV. For extraction of DNA, an aliquot of 400 μl EDTA-anticoagulated blood was collected and added with 900 μl distilled water to lyse red blood cells. Following removal of supernatant by centrifugation, 50 μl DNA Extraction Reagent was added and boiled for 10 min. Then 2 μl of supernatant was mixed with 40 μl PCR reaction solution and 3 μl of Taq polymerase. The further qRT-PCR reaction was performed with degeneration at 93°C for 2 min and the initial 10 cycles of 93°C for 45 sec and 55°C for 60 sec, and then 30 cycles of 93°C for 30 sec and 55°C for 45 sec. In each run, the provided negative control, positive control, and four calibrations (4-7 log_10 _copies/ml) for defining the standard curve, were also included. Calculations of *Ct*, preparation of standard curve and quantification of each sample's DNA were performed by the Option Monitor 2 Software. According to the manufacture's instructions, the detection limit of this qRT-PCR was 500 copies/ml with a *Ct *threshold of 30, hence those samples with their viral loads below the limit were interpreted as negative.

### Serological assay

Antibodies to HCMV were tested by enzyme-linked immunosorbent assay (ELISA) using commercial kits for IgM (Bell Biological Technology Co., Ltd., Beijing, China) and IgG (DIA.PRO Co., Italy). HCMV IgG AI was measured by the same IgG ELISA kit with some modifications [40]. In brief, the concentration of IgG for each sample was adjusted to 0.5 IU/ml-8.0 IU/ml by dilution, which was the most reliable range for detection in our laboratory. Diluted samples (50 μl) were added to each of the duplicate wells. Then one well was added with 50 μl specimen diluent (2% casein, 10 mM Tris-citrate buffer, pH 6.0, 0.1% Tween 20, 0.09% Na-azide, and 0.1% Kathon GC) while the other was added with 50 μl urea (8 M), followed by incubation at 37°C for 1 h. After washing for 5 times, the wells were incubated with 100 μl horseradish peroxidase-conjugated polyclonal antibodies to human IgG at 37°C for 1 h. Following 5 washes, the IgG was revealed by 100 μl chromogen/substrate (50 mM citrate-phosphate buffer, 4% dimethylsulphoxide, 0.03% TMB, and 0.02% H_2_O_2_). Optical densities were read at 450 nm. The AI was calculated as follows: (net OD in the presence of urea/net OD in the absence of urea) × 100%. In each test, control sera of high, intermediate, and low AI were included. Based on our previous study [40], an AI of less than 30% was considered as low, higher than 50% as high, and between 30% and 50% as moderate.

### Statistical analysis

With SPSS 13.0 statistical software, we performed chi-square analysis to compare difference between groups. The value of α was adjusted to 0.017 (0.05/3) by Bonferroni correction for multiple comparisons, otherwise it was set as 0.05. A p value < α was considered as statistically significant.

## Competing interests

The authors declare that they have no competing interests.

## Authors' contributions

SZ carried out the nested PCR and serological assays, analyzed the data, and drafted the manuscript. YHZ and YH initially conceived of the study, critically revised the experiment design and the manuscript. LL collected the samples and carried out the qRT-PCR. YH funded the study. All authors read and approved the final manuscript.
